# Characterization of a novel multidomain CE15-GH8 enzyme encoded by a polysaccharide utilization locus in the human gut bacterium *Bacteroides eggerthii*

**DOI:** 10.1038/s41598-021-96659-z

**Published:** 2021-09-03

**Authors:** Cathleen Kmezik, Daniel Krska, Scott Mazurkewich, Johan Larsbrink

**Affiliations:** 1 Division of Industrial Biotechnology, Department of Biology and Biological Engineering, Chalmers University of Technology, 412 96 Gothenburg, Sweden; 2 Wallenberg Wood Science Center, Chalmers University of Technology, 412 96 Gothenburg, Sweden

**Keywords:** Carbohydrates, Biochemistry, Enzymes, Hydrolases

## Abstract

Bacteroidetes are efficient degraders of complex carbohydrates, much thanks to their use of polysaccharide utilization loci (PULs). An integral part of PULs are highly specialized carbohydrate-active enzymes, sometimes composed of multiple linked domains with discrete functions—multicatalytic enzymes. We present the biochemical characterization of a multicatalytic enzyme from a large PUL encoded by the gut bacterium *Bacteroides eggerthii*. The enzyme, *Be*CE15A-Rex8A, has a rare and novel architecture, with an N-terminal carbohydrate esterase family 15 (CE15) domain and a C-terminal glycoside hydrolase family 8 (GH8) domain. The CE15 domain was identified as a glucuronoyl esterase (GE), though with relatively poor activity on GE model substrates, attributed to key amino acid substitutions in the active site compared to previously studied GEs. The GH8 domain was shown to be a reducing-end xylose-releasing *exo*-oligoxylanase (Rex), based on having activity on xylooligosaccharides but not on longer xylan chains. The full-length *Be*CE15A-Rex8A enzyme and the Rex domain were capable of boosting the activity of a commercially available GH11 xylanase on corn cob biomass. Our research adds to the understanding of multicatalytic enzyme architectures and showcases the potential of discovering novel and atypical carbohydrate-active enzymes from mining PULs.

## Introduction

The human gut microbiota (HGM) is characterized by very high cell densities of diverse microbial communities. One of its major roles is the degradation of recalcitrant dietary fiber and simultaneous secretion of short-chain fatty acids, which have been associated with numerous health benefits^1^. Understanding the HGM-host relationship is a major research field^2,3^ and the composition of the HGM is highly variable and influenced by factors such as diet, genetics, C-section vs. natural delivery, breastfeeding, gender, age, and medication^4^. Whether or not there is an “ideal” HGM composition is not fully known^5^, but in healthy adults the dominant bacterial phyla are typically Bacteroidetes and Firmicutes^6^. Both of these phyla include numerous species capable of efficiently degrading complex polysaccharides, which are a major component of dietary fiber.

To facilitate the complete degradation of recalcitrant dietary fiber, many Bacteroidetes species utilize polysaccharide utilization loci (PULs)^7^, which are discrete gene clusters encoding all proteins necessary to metabolize a specific polysaccharide^8,9^. The starch utilization system (Sus) from the anaerobic human gut symbiont *Bacteroides thetaiotaomicron* was the first PUL described and serves as a template for the identification and description of new PULs^10,11^. In addition to enzymes, carbohydrate-binding proteins, and a sugar sensor/regulatory protein, the Sus also encodes the proteins SusC and SusD; SusC is an integral outer membrane maltooligosaccharide transporter and SusD is a surface-tethered maltooligosaccharide-binding protein^12^. Homologs of SusC/D are found in all PULs and thereby enable the prediction of PULs from genomic sequences. Both characterized and putative PULs are collected in the database PULDB, which is part of the carbohydrate-active enzymes database CAZy (www.cazy.org;^13,14^). PULs encode sets of carbohydrate-active enzymes (CAZymes) with activities corresponding to their glycan target and consequently the number of CAZymes may vary greatly between different PULs. Several PULs have been characterized to date and have been found to target a wide range of homo- and heteroglycans such as plant hemicelluloses and pectins, crystalline chitin, fungal mannans, and algal polysaccharides^15–19^. Based on the co-localization of CAZyme-encoding genes targeting specific glycans, PULs can be used to assess the diversity of glycans in the natural environment as well as for the discovery of novel enzymes or enzyme architectures in Bacteroidetes species^20–22^.

*Bacteroides eggerthii* is a Bacteroidetes member that has been isolated from both human and fish feces^23,24^, indicating a successful adaptation to different host diets. While this bacterium has not been studied extensively to date, it has shown to be abundant in patients with type 2 diabetes^25^. *B. eggerthii* 1_2_48FAA is predicted to encode 39 PULs, and consequently a plethora of corresponding putative CAZymes^14^. Only a handful of enzymes from *B. eggerthii* have been characterized to date, including the heparinase Hep I^26^, the *endo*-xylanase *Be*Xyn5A^27^, and the “multicatalytic” arabinofuranosidase-feruloyl esterase *Be*GH43/FAE^28^. Multicatalytic enzymes contain multiple connected catalytic domains with discrete functions, and while only few have been characterized so far, multicatalytic enzymes often display synergistic activities between these domains. For example, the CAZymes CelA (N-terminal glycoside hydrolase family 9 (GH9) *endo*-cellulase and C-terminal GH48 *exo*-cellulase) from *Caldicellulosiruptor bescii*^29^, ChiA (N-terminal *exo*- and C-terminal *endo*-chitinase domain) from *Flavobacterium johnsoniae*^16,30^, and *Bo*CE6-CE1 (acetyl-feruloyl esterase) from *Bacteroides ovatus*^20^, have significantly enhanced substrate turnover capabilities as full-length enzymes compared to their individual catalytic domains. In some cases, intramolecular synergy has however not been observed for multicatalytic enzymes^20,31,32^. This might be caused by a lack of appropriate substrates, analytics, or appropriate reaction conditions, or simply that there is no synergy between the catalytic domains.

Of the predicted PULs in the genome of *B. eggerthii*, PUL 27 is one of the largest and it is anticipated to confer xylan degradation abilities to the bacterium based on its encoded CAZymes^13,14^. Two of the aforementioned characterized *B. eggerthii* enzymes, *Be*Xyn5A and *Be*GH43/FAE, are also encoded by this PUL, and both enzymes have been shown to act on complex xylan^27,28^. In addition to *Be*GH43/FAE, the PUL encodes one more putative multicatalytic enzyme comprising an N-terminal carbohydrate esterase family 15 (CE15) and a C-terminal GH8 domain. Characterized CE15 members to date are glucuronoyl esterases (GEs), which have the proposed role of cleaving ester linkages between lignin and (4-*O*-methyl)-d-glucuronate decorations of glucuronoxylan and glucuronoarabinoxylan (GAX) in lignin carbohydrate complexes (LCCs)^33–39^. LCCs confer strength and rigidity to the plant cell wall and represent major obstacles in industrial enzymatic biomass hydrolysis processes^40–43^. In contrast to CE15, various activities have been demonstrated in GH8 including chitosanase, cellulase, licheninase, *endo*-*β*-1,4-xylanase and reducing-end xylose-releasing *exo*-oligoxylanase (Rex) enzymes^13^. These enzyme activities are found in multiple CAZy families, apart from Rex activity which is unique to GH8. The first Rex was isolated from *Bacillus halodurans* C-125 and, while it showed no activity on xylan, it released xylose moieties from the reducing end of xylooligosaccharides (XOs) longer than xylobiose, with xylotriose being the preferred substrate^44^. The combination of a CE15 domain and a GH8 domain into one single enzyme suggests a common substrate for the two catalytic domains, similar to the recently studied GE-xylanase *Ck*Xyn10C-GE15A from the hyperthermophilic bacterium *Caldicellulosiruptor kristjanssonii*, which additionally incorporates five carbohydrate-binding modules (CBMs)^32^. The polyspecificity of GH8 however precludes conclusive functional prediction of the *B. eggerthii* enzyme as a GE-xylanase fusion.

Here, our aim was to characterize the atypical multicatalytic enzyme from *B. eggerthii* comprising a CE15 and a GH8 domain to gain insight into its biological role. This enzyme architecture was found to be extremely rare, with the few identifiable homologs existing in the Bacteroidetes phylum. Biochemical characterization of the CE15 domain showed that it was active on standard GE substrates, though only with minor activity. This low activity was attributed to an amino acid substitution close to the catalytic serine, though changing the residue to the most conserved amino acid within the broader family did not increase activity. Assays on a wide range of substrates revealed the C-terminal GH8 domain to be a Rex, and the full-length protein was named *Be*CE15A-Rex8A. Direct synergy between the two catalytic domains could not be observed on GAX-rich corn cob biomass, possibly attributable to the minimal GE activity, though the Rex domain was able to boost the activity of a GH11 xylanase.

## Results and discussion

### Sequence based analysis

**Figure 1:**
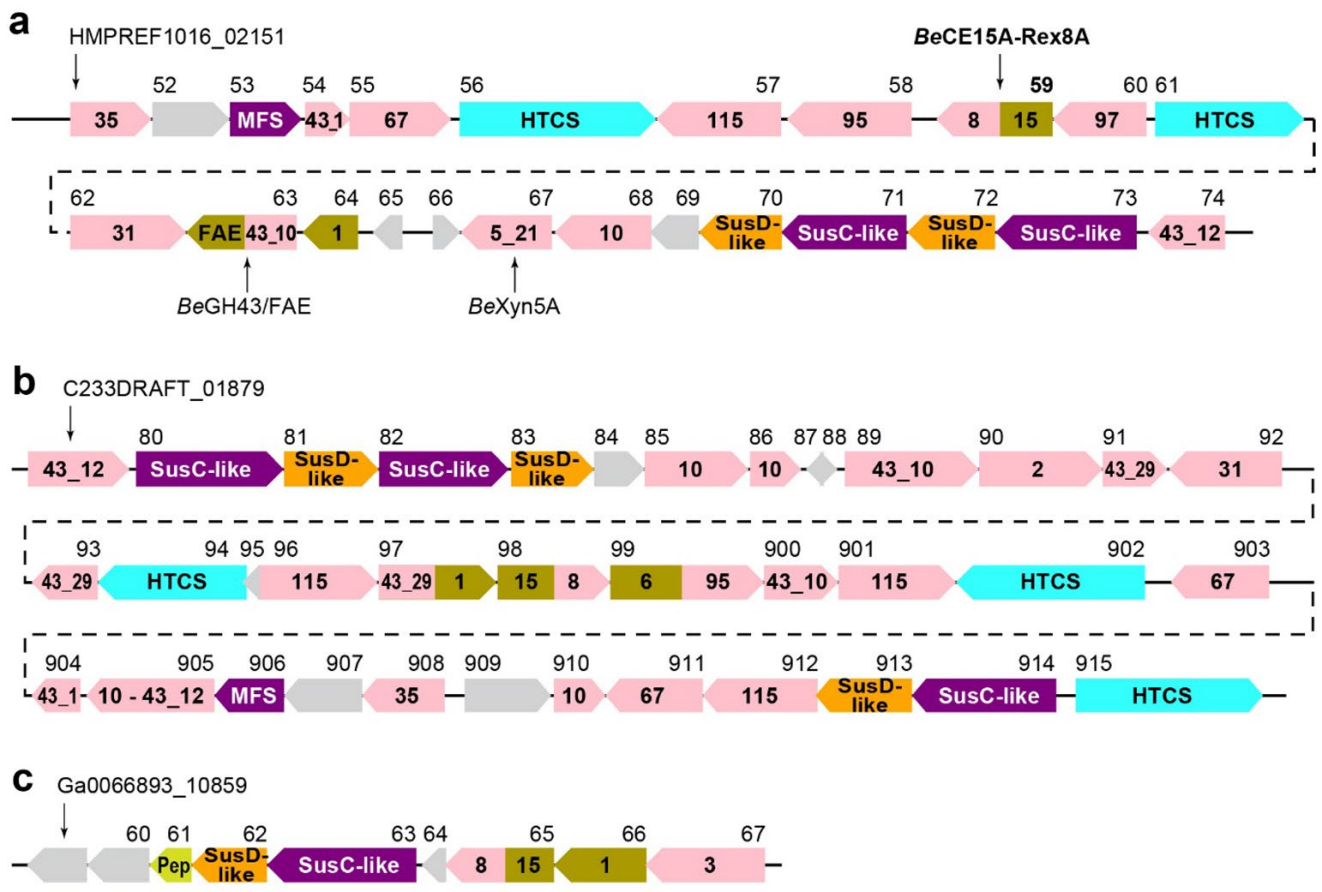
Overview of PULs containing CE15-GH8 enzymes as predicted by the PULDB^14^. **(a)** PUL 27 of *B. eggerthii*, **(b)** PUL 20 of *B. gallinarum*, and **(c)** PUL 6 of *Prevotella* sp. BP1-145 (identical to PUL 1 of *Prevotella* sp. BP1-148). Locus tags are shown above each corresponding gene, drawn to scale. Enzymes are shown with enzyme family numbers indicated: glycoside hydrolases in pink, carbohydrate esterases in brown, sugar transporters in purple (MFS—major facilitator superfamily), putative regulators in blue (HTCS—hybrid two-component system), peptidase in yellow-green (Pep), and proteins of unknown function in gray. Intergenic regions are shown with dashed lines and are not drawn to scale (283 bp between HMPREF1016_01261 and 01262, 30 bp between C233DRAFT_01892 and 01893, and 0 bp between C233DRAFT_01903 and 01904).

The 39 predicted PULs of *B. eggerthii* 1_2_48FAA range from solitary SusC/D pairs to loci spanning more than 30 genes. PUL 27 spans 24 genes (locus tags HMPREF1016_02151—HMPREF1016_02174; Fig. 1a)^14^. Curiously, no gene corresponding to HMPREF1016_02158 was listed in the PULDB. Translation of the intergenic sequence between HMPREF1016_02157 and HMPREF1016_02159 revealed a putative GH95 domain (785 amino acids) which is in agreement with enzymes found in previously studied PULs targeting GAX^15^. The collective enzyme repertoire of PUL 27, in addition to the previously characterized *Be*GH43/FAE (HMPREF1016_02163) and *Be*Xyn5A (HMPREF1016_02167), strongly supports the hypothesis of the PUL targeting complex glycans, with putative xylanase (GH10), *β*-xylosidase or *α*-l-arabinofuranosidase (GH43), *α*-glucuronidase (GH67, GH115), *α*-xylosidase (GH31), *α*-galactosidase (GH95, GH97) and feruloyl or acetyl esterase (CE1) activities (Table S1)^45–47^. No PUL with a similar architecture was found in the PULDB^14^.

The product of HMPREF1016_02159 in PUL 27 has a very unusual enzyme architecture, encoding a predicted multicatalytic enzyme comprising an N-terminal CE15 domain and a C-terminal GH8 domain, and a very short potential linker region. When compared to sequences in the NCBI protein database, a similar architecture was only found in three other species from the *Bacteroides* genus (*Bacteroides* sp. NSJ-48, *B. stercoris*, and *B. gallinarum*), each encoding one uncharacterized protein with 89–94% sequence identity (100% seq. coverage) to the *B. eggerthii* enzyme^46,48^. Furthermore, two homologs with lower similarity were found encoded by the more distantly related *Prevotella* sp. BP1-148 and *Prevotella* sp. BP1-145 (55% seq. id., 97% seq. coverage). Of these, only the *B. gallinarum* enzyme is found in a very large PUL likely targeting xylan (encoding enzymes from e.g. GH10, GH43, GH67, GH115, CE1; Fig. 1b), and the *Prevotella* enzymes are encoded by two identical small PULs that in addition to the CE15-GH8 enzyme only encode CAZymes from GH3 and CE1 (Fig. 1c)^14^. The CE15-GH8 architecture thus appears confined to the Bacteroidetes phylum and is strongly suggested to be involved in xylan turnover.

The individual domains of the *B. eggerthii* enzyme were compared to characterized enzymes from CE15 and GH8, respectively. The CE15 domain was most similar to *Ot*CE15B from the soil bacterium *Opitutus terrae* (seq. id. 44%, coverage 97%)^37^. *Ot*CE15B and the here investigated CE15 domain were phylogenetically more closely related to characterized fungal GEs than to other characterized GEs of bacterial origin and both contained a key disulfide bridge locking the catalytic serine and histidine in place as it is common in fungal GEs (Fig. S1). The catalytic triad was found to be conserved in *Be*CE15A (Ser230, Glu253, His357).

**Figure 2:**
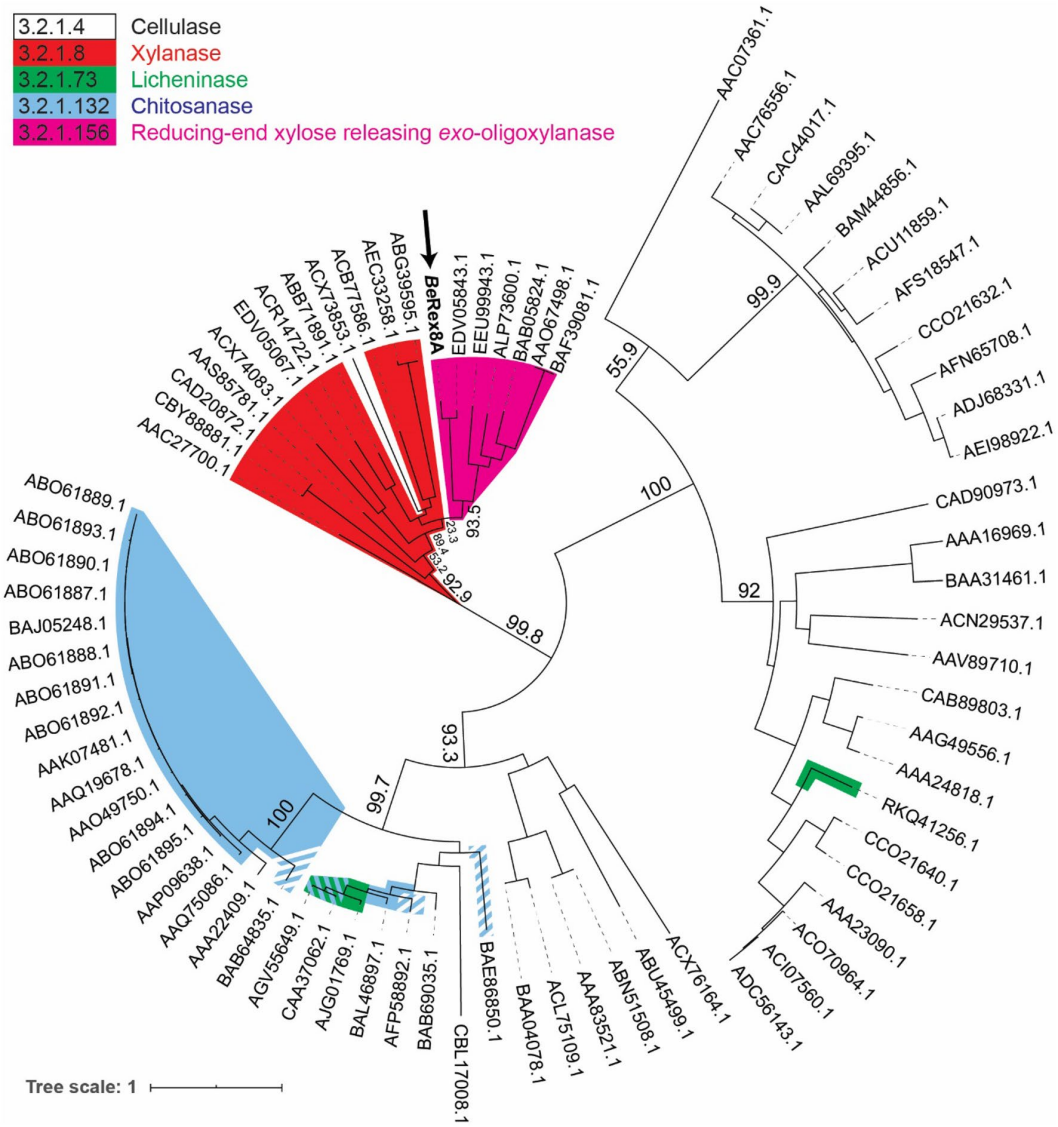
Phylogenetic tree of biochemically characterized GH8 domains. Proteins are labelled with Genbank accession numbers. The C-terminal Rex domain of *Be*CE15A-Rex8A is indicated in bold and marked with an arrow. Branches are colored by activity, with xylanase in red, Rex in magenta, licheninase in green, chitosanase in blue, and cellulase uncolored. Branches representing enzymes with dual specificity are striped with the corresponding colors.

In contrast to CE15, many more members belonging to GH8 have been biochemically characterized^13^. Previous work has shown phylogeny to be a useful tool to predict enzyme specificities in GH8 using a limited number of sequences^49^. As the number of characterized GH8 members have since grown significantly, we constructed a new phylogenetic tree using the catalytic domains of all characterized members of GH8 as well as the *B. eggerthii* GH8 domain (Fig. 2). The tree was largely in agreement with the previous one, with different specificities mostly clustering into separate clades, including a clade encompassing all xylanases characterized to date. Rex enzymes formed a separate branch within the xylanase clade. The *B. eggerthii* GH8 domain was found to be most similar to *Bi*Rex8A from *Bacteroides intestinalis*^50^; the enzymes share 84% sequence identity which strongly indicates a similar function. *Bi*Rex8A was characterized simultaneously with *Bi*Xyn8A from the same organism, where the latter was shown to be an *endo*-xylanase, as *Bi*Xyn8A hydrolyzed both wheat arabinoxylan and oat spelt xylan into XOs^50^. *Bi*Rex8A on the other hand showed no xylanase activity but was instead able to release xylose moieties from the reducing end of XOs. The same study demonstrated that both *Bi*Rex8A and *Bi*Xyn8A shared the same conserved catalytic residues^50^, which are also conserved in the *Be*Rex8A domain (Glu483, Asp541 and Asp679; Fig. S2).

### Biochemical characterization of the *Be*CE15A-Rex8A CE15 domain

**Figure 3:**
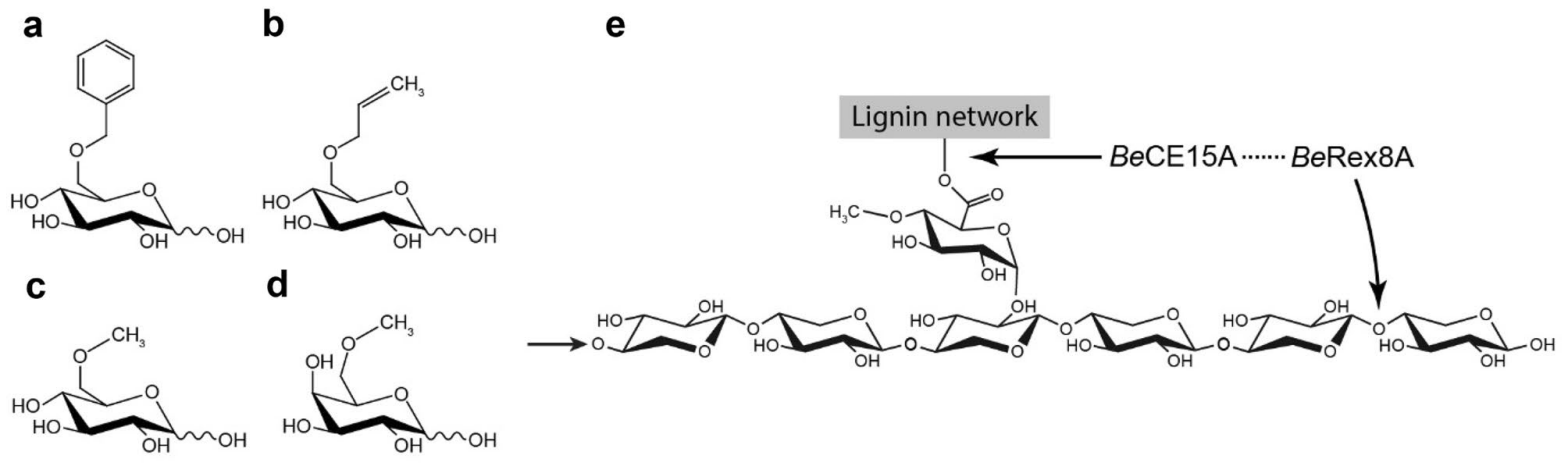
Substrates used to assay glucuronoyl esterase activity of *Be*CE15A: **(a)** benzyl glucuronoate, **(b)** allyl glucuronoate, **(c)** methyl glucuronoate, and **(d)** methyl galacturonoate. **(e)** The suggested target of the full-length *Be*CE15A-Rex8A enzyme, consists of a xylooligosaccharide (or longer xylan chain as indicated by the small arrow) decorated with GlcA, which is further ester-linked to lignin or lignin fragments.

**Figure 4:**
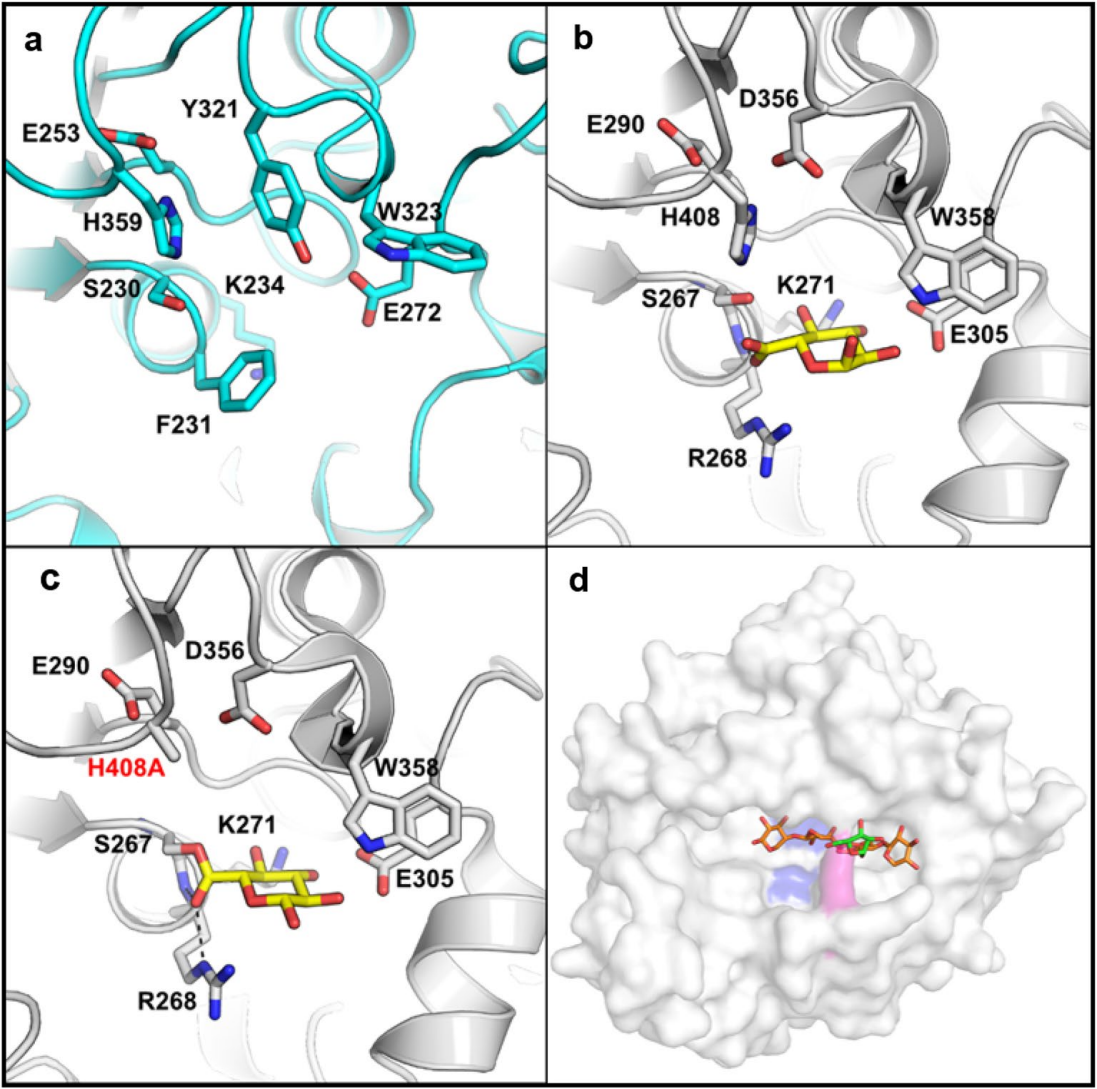
Active site of a homology model of *Be*CE15A and model structure of *Be*Rex8A. **(a)** A homology model of the *Be*CE15A was generated with Phyre2^56^ using the CE15 from *Hypocrea jecorina* (PDB ID: 3PIC) as a template and compared to structures of **(b)** the wild type *Ot*CE15A (PDB ID: 6SYR) in complex with glucuronate (yellow sticks) and **(c)** a H408A variant of *Ot*CE15A (PDB ID: 6SZ4) which was trapped with glucuronate covalent adduct and shows the interaction with the usually conserved active site arginine. The equivalent position in *Be*CE15A is predicted to be a phenylalanine (Phe231). **(d)** The model structure of *Be*Rex8A was generated by Phyre2^56^ and based on *Pb*Rex8A (PDB ID: 6TRH). The catalytic residues are shown in blue. The Arg670 residue is shown in purple. The arabinoxylooligosaccharide from *Pb*Rex8A is modelled into the active site. The figure was made using PyMOL 2.3.

To confirm the putative functions of *Be*CE15A-Rex8A, the enzyme was heterologously produced in *E. coli* both as a full-length enzyme (91.5 kDa) and as the individual domains *Be*CE15A (46.8 kDa; amino acid residues 32-413) and *Be*Rex8A (50.0 kDa; amino acid residues 414—812). *Be*CE15A-Rex8A and *Be*CE15A were assayed on the standard GE substrates benzyl glucuronoate (BnzGlcA), allyl glucuronoate (AllylGlcA), methyl glucuronoate (MeGlcA) and methyl galacturonoate (MeGalA) (Fig. 3). In contrast to previously studied GEs, none of the reactions were saturable up to concentrations of 40 mM substrate, precluding determination of either *k*_*cat*_ or *K*_M_ parameters. However, the catalytic efficiency (*k*_*cat*_*/K*_M_) could be determined using linear regression and showed that *Be*CE15A-Rex8A and *Be*CE15A were most active on BnzGlcA, with the activity decreasing successively on AllylGlcA, MeGlcA, and MeGalA (Table 1). This is in accordance with other characterized bacterial GEs, and consistent with the hypothesis that GEs prefer bulky substrates that are ester-linked to the *O*-6 position of a uronic acid moiety^37,51,52^, mimicking lignin or a lignin fragment in LCCs. In GEs, the rate-limiting step has been proposed to be the deacylation of the acyl-enzyme intermediate, given the similar *k*_*cat*_ values determined for various enzymes to date^52^. The low *k*_*cat*_/*K*_M_ values of *Be*CE15A may thus be a result of high *K*_M_ values, indicating a poor fit of the model substrates in the active site. The isolated *Be*CE15A was approximately as active on the model substrates as the full-length enzyme, with roughly equal catalytic efficiencies on BnzGlcA, and 1.5-fold higher catalytic efficiency on AllylGlcA. This indicates that the truncation of *Be*CE15A-Rex8A into *Be*CE15A did not negatively affect the GE. The observed catalytic efficiencies were minimal compared to the majority of previously studied GEs reported in literature and the activity on BnzGlcA was approximately 500-fold lower than that of *Tt*CE15A from *Teredinibacter turnerae*, which to date has the highest reported *k*_*cat*_/*K*_M_ value for this substrate^51^. However, enzymes with even lower *k*_*cat*_/*K*_M_ values on BnzGlcA than *Be*CE15A have previously been studied, including the most closely related characterized enzyme *Ot*CE15B from *O. terrae* (*k*_*cat*_/*K*_M_ value of 18.6 s^−1^ M^−1^), which is approximately fourfold lower than that of *Be*CE15A^37^. *Ot*CE15B is an exception among studied GEs, as it has a tyrosine residue in the equivalent position of the conserved active site arginine residue believed to partake in forming the oxyanion hole and stabilizing the transition state during catalysis^37,52,53^. *Be*CE15A has an unexpected non-polar phenylalanine residue (Phe231) in equivalent position, which would not be able to electrostatically stabilize the transition state with its side chain (Fig. 4). To investigate whether replacement of this residue with the expected arginine residue would improve the activity on GE model substrates, we constructed an F231R variant of *Be*CE15A. Instead of increasing the activity, the result was however a complete loss of GE activity. Similarly, a substitution with a tyrosine (F231Y), as present in *Ot*CE15B, also led to a complete loss of GE activity (data not shown).

**Table 1:** Activity of *Be*CE15-Rex8A and *Be*CE15A on GE model substrates.

Enzyme	Substrate	*k*_*cat*_/*K*_M_ (s^−1^ mM^−1^)
*Be*CE15A*-*Rex8A	BnzGlcA	0.0693 ± 0.0009
	AllylGlcA	0.0159 ± 0.0005
	MeGlcA	Trace
	MeGalA	Trace
*Be*CE15A	BnzGlcA	0.0753 ± 0.0008
	AllylGlcA	0.0235 ± 0.0002
	MeGlcA	0.0075 ± 0.0003
	MeGalA	0.0008 ± 0.0002

“Trace” stands for trace activity detected for 0.02 µM min^−1^ mg^−1^ protein. The reactions were not saturable up to 40 mM and linear regression was used to calculate the *k*_cat_/*K*_M_ values using GraphPad Prism 8. The results are based on triplicate measurements and presented with standard errors of the mean. The *Be*CE15A variants F231R and F231Y were also assayed, but no activity could be detected.

### Biochemical characterization of the *Be*CE15A-Rex8A GH8 domain

**Figure 5:**
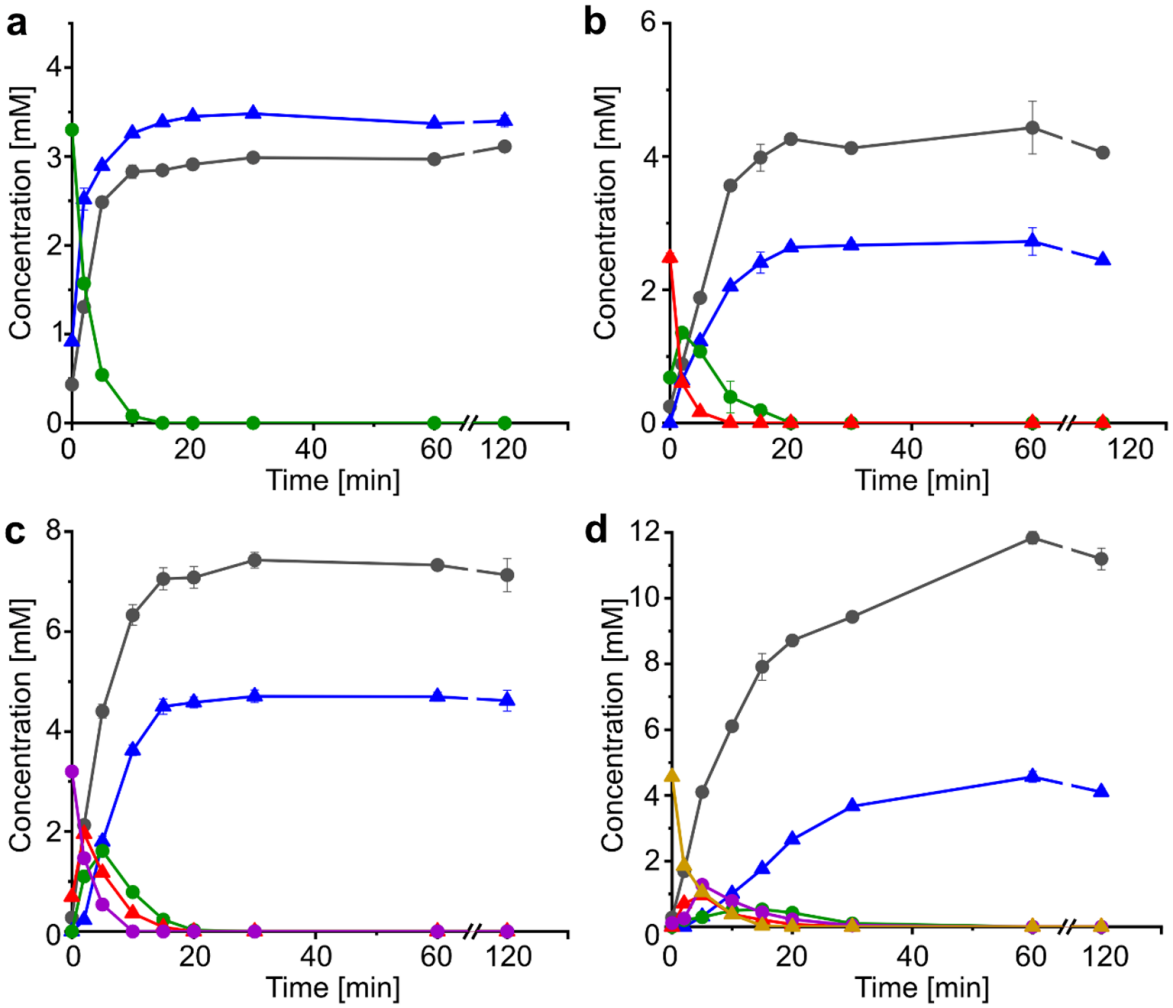
Hydrolysis of xylooligosaccharides by *Be*Rex8A. Substrates used were **(a)** xylotriose, **(b)** xylotetraose, **(c)** xylopentaose, and **(d)** xylohexaose. Concentrations are shown for xylose (gray circle), xylobiose (blue triangle), xylotriose (green circle), xylotetraose (red triangle), xylopentaose (purple circle), and xylohexaose (golden triangle). Data are presented as averages of triplicate experiments with standard errors of the mean.

As GH8 is a polyspecific family, the GH8 domain of *Be*CE15A-Rex8A was assayed on a range of polysaccharides: cellulose, birchwood and beechwood xylan, wheat arabinoxylan, linear ivory nut mannan, mixed linkage *β*-glucan from barley, as well as starch. No activity could be detected on any of these substrates even after prolonged incubations. Previously studied Rex enzymes have been shown to either be inactive or have minimal activity on polymeric xylan and instead are active on XOs^44,49,50,54,55^. Similarly, *Be*Rex8A was able to hydrolyze XOs ranging from xylotriose (X_3_) to xylohexaose (X_6_), and only trace activity (above 0.02 µM min^−1^ mg^−1^ protein) was observed on X_2_ when incubated for prolonged periods of time (Fig. 5). X_1_ and X_2_ were the end products of all reactions. Our time-course analysis shows highly similar hydrolysis progress curves to those of *Bi*Rex8A from *Bacteroides intestinalis*^50^, with the substrates being sequentially shortened into intermediate products, themselves acting as new substrates, and with a concomitant accumulation of X_1_ and X_2_ as end products (Fig. 5). *Be*Rex8A was not active on *p*NP-xylobiose or borohydrate-reduced xylotriose, further supporting the Rex activity.

Attempts to crystallize and determine the structure of *Be*CE15A-Rex8A or its parts were unfortunately not successful. However, modeling of *Be*Rex8A using Phyre2^56^ yielded a predicted protein structure with 95% coverage and 100% confidence based on the structurally determined E70A variant of *Pb*Rex8A from *Paenibacillus barcinonensis* (PDB ID: 6TRH; 42% seq. id. to *Be*Rex8A; Fig. 4d)^57^. *Pb*Rex8A was previously shown to have minimal activity on xylan and a loop comprised of Leu320-His321-Pro322 blocking the active site after the + 1 subsite was attributed to the inability of the enzyme to efficiently bind polysaccharides and generate products longer than xylose^57^. Attempts to reduce the size of the loop to open up the active site did however not improve the activity on xylan^57^. An equivalent to the Leu320-His321-Pro322 loop in *Pb*Rex8A is not found in *Be*Rex8A, but the modeled structure shows a similar active site groove, blocked by a single arginine residue (Arg670; Fig. 4d). This arginine residue appeared to form a tunnel allowing access of unsubstituted oligo- or polysaccharides. We hypothesized that this residue may play a role in the specificity for shorter oligos, and the lack of activity on polysaccharide chains, and constructed an R670A variant. However, this variant was inactive on polymeric xylan, similar to the wild type enzyme (data not shown). Deeper structural investigation would be needed to shed light on possible interchangeability between Rex vs. *endo*-xylanase activity.

### Boosting of xylanase hydrolysis of corn cob

**Figure 6:**
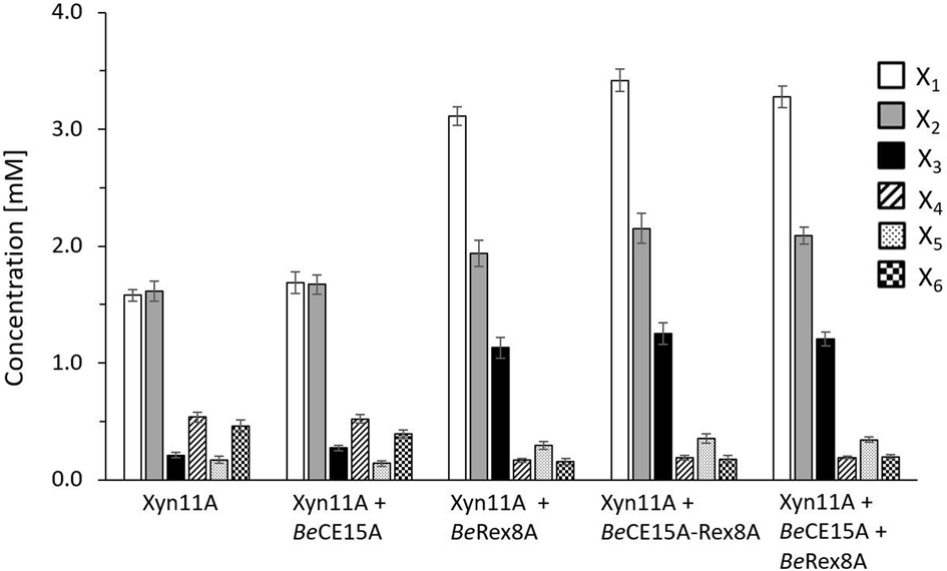
Xylooligosaccharide production profiles from corn cob biomass hydrolysis. The *endo*-*β*-1,4-xylanase Xyn11A from *Neocallimastix patriciarum* was either incubated alone or supplemented with *Be*CE15A, *Be*Rex8A, *Be*CE15A-Rex8A or an equimolar mix of *Be*CE15A and *Be*Rex8A. Reactions in which *Be*CE15A, *Be*Rex8A, and *Be*CE15A-Rex8A were incubated without Xyn11A yielded no detected sugars (data not shown), as expected of a Rex enzyme and a GE. Presented are xylose (X_1_, white), xylobiose (X_2_, gray), xylotriose (X_3_, black), xylotetraose (X_4_, striped), xylopentaose (X_5_, dotted), and xylohexaose (X_6_, checkered) concentrations after 30 h of incubation that were determined using high-performance anion-exchange chromatography with pulsed amperometric detection (HPAEC-PAD). Data are shown as average of triplicate experiments with standard errors of the mean.

Enzymes present in PULs are expected to act in concert in the degradation of a specific polysaccharide. Based on the GE and Rex activities of the individual catalytic domains of *Be*CE15A-Rex8A, the enzyme was expected to aid in the degradation of complex xylans of the plant cell wall. The reason for combining activities presumed to target complex LCCs and shorter XOs into one single enzyme is however not clear. To gain further insight into the function of *Be*CE15A-Rex8A, as well as its truncated single domain versions (*Be*CE15A and *Be*Rex8A), the enzymes were assayed for their ability to boost the action of a commercially available GH11 xylanase (Xyn11A), which has previously been used successfully in similar experiments^20,31^. Ball-milled corn cob biomass, which has a high content of GAX^15^, was used as substrate. No release of sugars was observed when no enzyme was added (data not shown), and similarly no released sugars were detected if *Be*CE15A-Rex8A, *Be*CE15A or *Be*Rex8A were added without Xyn11A (data not shown). Addition of Xyn11A (control reaction) lead to the release of small amounts of XOs ranging from X_1_ to X_6_ (Fig. 6). The main products were X_1_ and X_2_ with concentrations reaching 1.6 mM each after 30 h, and substantially more X_4_ and X_6_ were released than X_3_ and X_5_. Supplementation of Xyn11A with *Be*CE15A did not alter XO release substantially compared to the control reaction. Supplementation of Xyn11A with *Be*Rex8A, *Be*CE15A-Rex8A or an equimolar mix of *Be*CE15A and *Be*Rex8A increased X_1_ (twofold), X_2_ (1.3-fold), X_3_ (5.6-fold) and X_5_ concentrations (twofold), while X_4_ and X_6_ concentrations were reduced to roughly a third compared to the control reaction. The total xylose equivalents from X_1_-X_6_ that were released by Xyn11A when supplemented with *Be*Rex8A increased 20–30% compared to the reaction of Xyn11A alone, and do not appear to stem simply from conversion of longer XOs to short ones by the Rex enzyme. Possibly, the apparent improvement of Xyn11A could be a result of reduced product inhibition.

The reason for the inability of *Be*CE15A to boost xylanase activity on corn cob biomass is not clear but echoes the results of the CE15 domain from the *Caldicellulosiruptor kristjanssonii* encoded *Ck*Xyn10C-GE15A, which similarly did not appear to boost xylanase activity directly either with commercial enzymes or the linked *Ck*Xyn10C xylanase domain^32^. Possibly, the effect of GEs on xylanases cannot be monitored by sugar release measurements due to the overall complexity of the (un-pretreated) material and reduced access to LCC esters that the enzymes can target. Alternatively, *Be*CE15A, with its atypical active site residues, may be more specialized to target structures that were not present or accessible in the here utilized corn cob biomass. The low activity of *Be*CE15A on GE model substrates, similar to that of *Ot*CE15B^37^, suggests that these enzymes might have a different role in biomass turnover than other so far characterized CE15 enzymes. The main activity of characterized CE15 members to this date has been (4-*O*-methyl)-glucuronoyl esterase activity^13^, but the incorporation of a CE15 enzyme into a PUL suggests that the activity of *Be*CE15A supports xylan degradation. Deeper investigation of atypical enzymes such as *Be*CE15A and *Ot*CE15B holds the potential of adding to our knowledge on enzymatic biomass degradation and might be an interesting target for the improvement of industrial enzyme cocktails.

**Figure 7:**
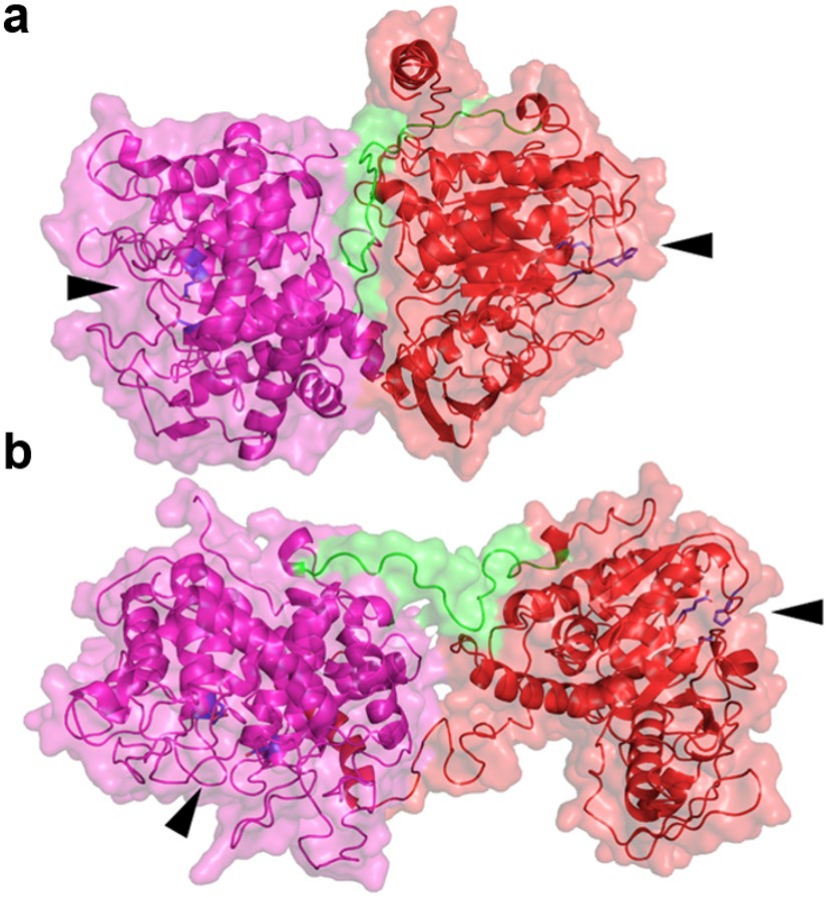
Full length model of *Be*CE15A-Rex8A using I-TASSER^58^ **(a)**, and Phyre2^56^ **(b)**. In both models, the GE domain is colored red, the Rex domain is purple, the potential linker region is green, and the active-site residues are blue. In both models the active sites of the two domains are positioned facing away from each other and marked by black arrows.

Comparing supplementation of Xyn11A with the full-length *Be*CE15A-Rex8A and an equimolar mix of its single domains *Be*CE15A + *Be*Rex8A did not reveal significant differences in the XO production profiles over the whole course of the experiment (Fig. S3). Deducing the preferred substrate of a multicatalytic enzyme can be challenging due to the highly specialized nature of these proteins and the vast diversity among polysaccharides, especially in the context of the complex cell wall polymer network. A lack of intramolecular enzyme synergy has also been observed for other multicatalytic enzymes, such as *Fj*CE6-CE1 from *F. johnsoniae*^20^, *Ck*Xyn10C-GE15A from *C. kristjansonii*^32^, and *Dm*CE1B from *Dysgonomonas mossii*^31^. Given the complexity of the substrates targeted by these enzymes, which are presumed to be part of LCCs, it is currently unclear whether the lack of observed intramolecular enzyme synergy is the result of missing intramolecular synergy, a lack of the right substrate, or another unknown reason. Typically, multicatalytic enzymes are joined by flexible linkers of varying length or small domains^29,32^. In *Be*CE15A-Rex8A, a short potential linker is present between Trp402 and Ala423, although exactly how flexible the linker is remains unclear. While no experimental structural data is available, multiple models constructed using the Phyre2^56^ and I-TASSER^58^ structural modelling servers suggest that the catalytic domains may be in close contact with each other (Fig. 7). Additionally, the domains appear to be oriented with their active sites facing in opposite directions. Whether the active sites are able to act in close proximity or not, depending on the length and flexibility of the putative linker, is currently unclear and would need support with structural data.

Analysis using Signal P 5.0^59^ identified a 23 amino acid long signal peptide with 95% likelihood as Sec/SPI, indicating that the protein is likely secreted into the periplasm, but whether *Be*CE15A-Rex8A is further transported outside the cell is not known. The presumed biological role of GEs would indicate that the target substrate(s) of *Be*CE15A is found in large LCCs that are unlikely to be imported into the periplasm. Conversely, the Rex activity of *Be*Rex8A would be more in keeping with how final degradation of poly- and oligosaccharides in PULs is believed to mainly occur in the periplasm to prevent “leakage” of metabolizable sugars to surrounding cells^8,9,18,19^. The low GE activity of the enzyme and atypical active site setup might indicate that GlcA in xylan can be esterified with as of yet unidentified moieties that are hydrolyzed in the periplasm by *B. eggerthii*. Identification of such motifs would likely require significant efforts, though enzymes such as *Be*CE15A and *Ot*CE15B could be highly useful tools in such an endeavor.

## Conclusion

In this study we biochemically characterized the multicatalytic enzyme *Be*CE15A-Rex8A. The N-terminal domain was identified as a GE having minimal activity on model substrates and harboring a highly unusual amino acid substitution close to the catalytic serine that might play an important role in substrate turnover or substrate preferences that are yet unidentified in CE15. The C-terminal domain was identified as a Rex, an activity that has been demonstrated in very few enzymes to date. The here described enzyme architecture of *Be*CE15A-Rex8A was shown to be very rare and confined to a few PULs within the bacterial phylum of Bacteroidetes. This work further highlights the usefulness of mining PULs for the discovery of novel enzyme types and architectures.

## Material and methods

### Phylogenetic tree

The amino acid sequences of GH8 enzymes listed as characterized were downloaded from CAZy (Nov 2020), trimmed to only contain the catalytic domains, and subsequently aligned using MUSCLE^60^. The phylogenetic tree was built based on the alignment using IQ-TREE^61^, with automatic finding of the best substitution model (LG + F + I + G4) and 1000 ultrafast bootstraps. The maximum-likelihood tree was visualized using iTOL^62^.

### Cloning of *Be*CE15A-Rex8A and protein variants

The putative *Be*CE15-GH8 was amplified from genomic DNA of *B. eggerthii* 1_2_48FAA by PCR (primers listed in Table S2) and the products cloned into a modified pET-28a vector, by ligation independent cloning (In-Fusion HD kit; Clontech Laboratories), containing an N-terminal His_6_ tag and a tobacco etch virus protease cleavage site. A signal peptide predicted at the N-terminal end of the gene encoding *Be*CE15A-Rex8A (residues 1–31) was not included for protein production. Enzyme variants were created by site-specific mutagenesis by the QuikChange method using the primers listed in Table S2^63^.

### Protein production and purification

Cell cultures harboring expression vectors were grown in lysogeny broth at 37 °C and 180 rpm until cells reached mid-log phase (OD_600_ 0.4–0.6), at which point protein production was induced by addition of 0.2 mM isopropyl-*β*-d-1-thiogalactopyranoside, and cells cultured overnight (16 °C and 180 rpm). The cells were harvested by centrifugation and lysed by sonication. The resulting protein containing crude lysate was purified using immobilized metal ion affinity chromatography as previously described^37^. Purified protein was concentrated and buffer exchanged (*Be*CE15A in 50 mM Tris pH 8.0 + 100 mM NaCl; *Be*Rex8A in sodium phosphate pH 6.5 + 100 mM NaCl; and *Be*CE15A-Rex8A in 50 mM Tris pH 8.0 + 250 mM NaCl + 5% w/v glycerol) using 10 kDa cut-off centrifugal filter units (Amicon Ultra-15, Merck-Millipore) and imidazole concentrations were reduced to < 1 mM. Sodium dodecyl sulfate polyacrylamide gel electrophoresis using Mini-PROTEAN TGX Stain-Free Gels (BIO-RAD) was used to verify molecular weight and protein purity. Protein concentrations were determined using a Nanodrop 2000 Spectrophometer (Thermo Fisher Scientific) using extinction coefficients and molecular weights predicted by Benchling.

### Biochemical characterization of the CE15 domain

pH dependency was established for *Be*CE15A by comparing the activity on BnzGlcA in a range of buffers and pH values (Fig. S4). A pH dependency profile for *Be*Rex8A could not be established as the enzyme fell out of solution at pH values different than 6.5 ± 0.5. Assays on model GE substrates (BnzGlcA, AllylGlcA, MeGlcA, MeGalA) were performed at pH 7.5 for comparison to other GEs as previously described^32,37^ using the d-Glucuronic/d-Galacturonic Acid Assay Kit (Megazyme). Briefly, concentrations of substrate up to 40 mM were incubated with *Be*CE15A at room temperature in a coupled enzyme assay with uronate dehydrogenase and the formation of nicotinamide adenine dinucleotide hydride was monitored at 340 nm. Data were analyzed using GraphPad Prism 8.4.2, and *k*_cat_/*K*_M_ values were determined by linear regression.

### Biochemical characterization on XOs and complex substrates

All here described substrates were purchased from Megazyme if unless stated otherwise. Reactions were incubated at 37 °C with mixing at 500 rpm and contained *Be*Rex8A (2 µM; in 50 mM sodium phosphate buffer pH 6.0 + 100 mM NaCl) and the different substrates. Screening of possible polysaccharide hydrolyzing ability of the Rex8A domain was done using 1.25% w/v cellulose, birchwood xylan, beechwood xylan (Apollo scientific), wheat arabinoxylan, linear ivory nut mannan, mixed linkage *β*-glucan from barley, or starch, with sugar release monitored using the dinitrosalicylic acid assay. Xylooligosaccharides tested were xylobiose (X_2_; 3.2 mM), xylotriose (X_3_; 3.25 mM), xylotetraose (X_4_; 3.3 mM), xylopentaose (X_5_; 2.65 mM) and xylohexaose (X_6_; 3.33 mM). Samples were flash-frozen in liquid nitrogen, diluted with HCl (0.1 M final concentration) to stop the enzymatic reaction and analyzed using HPAEC-PAD (see below).

Corn cob biomass for xylanase hydrolysis studies was produced by processing corn cob (excluding corn grains) in a kitchen blender followed by ball-milling into a fine powder, washing with water, and then freeze-drying. The corn cob was used as substrate (0.45% w/v) with *Be*CE15A-Rex8A, *Be*CE15A or *Be*Rex8A, incubated at 37 °C and 1000 rpm in 100 mM sodium phosphate pH 6.5 including 0.5 µM of each enzyme, in various combinations with and without addition of the commercially available *endo*-*β*-1,4-xylanase Xyn11A from *N. patriciarum* (E-XYLNP; Megazyme; concentration in assay 11 µM). The samples were flash-frozen in liquid nitrogen and stopped by addition HCl (0.1 M final concentration) before being analyzed using HPAEC-PAD.

### High-performance anion-exchange chromatography with pulsed amperometric detection

HPAEC-PAD was performed on a Dionex ICS-5000 + (Thermo Fisher Scientific) equipped with a Dionex CarboPac™ PA200 column (Thermo Fisher Scientific). To achieve sufficient separation of the XOs a constant flow of 0.5 mL/min and a multistep gradient (Table S3) were applied using deionized water, 300 mM NaOH, and 1 M NaAc. Prior to use dissolved oxygen was removed from all solutions by sparging with helium gas.

### Structural models of *Be*CE15A-Rex8A

The model for *Be*Rex8A was generated with Phyre2^56^ and based on the structurally determined E70A variant of *Pb*Rex8A from *P. barcinonensis*. Models of full-length *Be*CE15A-RexA domains combined were generated both with the Phyre2 server^56^ and with the I-TASSER server^58^. When selecting a model from I-TASSER, manual inspection of the predicted folding of the individual domains was used (in comparison to crystal structures of other Rex and GE domains) in order to select the most likely model.

## Supplementary Information

Supplementary Information.

## Abbreviations

AllylGlcA: Allyl glucuronic acid ester
BnzGlcA: Benzyl glucuronoate
CAZy: Carbohydrate-active enzymes database
CAZyme: Carbohydrate-active enzyme
CE: Carbohydrate esterase
CE15: Carbohydrate esterase family 15
GAX: Glucuronoarabinoxylan
GE: Glucuronoyl esterase
GH: Glycoside hydrolase
HGM: Human gut microbiota
HPAEC-PAD: High-performance anion-exchange chromatography with pulsed amperometric detection
LCC: Lignin-carbohydrate complex
MeGalA: Methyl galacturonoate
MeGlcA: Methyl glucuronoate
PDB: Protein data bank
PUL: Polysaccharide utilization locus
PULDB: Polysaccharide-Utilization Loci DataBase
Rex: Reducing-end xylose-releasing *exo*-oligoxylanase
Sus: Starch utilization system
XO: Xylooligosaccharide
X_1-6_: Xylose, xylobiose, xylotriose, xylotetraose, xylopentaose and xylohexaose, respectively
