# Supplementary material for: Characterization of a novel multidomain CE15-GH8 enzyme encoded by a polysaccharide utilization locus in the human gut bacterium Bacteroides eggerthii
Source: Sci Rep. 2021 Sep 3;11:17662. doi: 10.1038/s41598-021-96659-z (PMC8417218; doi:10.1038/s41598-021-96659-z)
Supplement: Supplementary file 1 — Supplementary Information. [file 41598_2021_96659_MOESM1_ESM.pdf]

Supplemental Information

Characterization of a novel multidomain CE15-GH8 enzyme encoded by a polysaccharide utilization locus in the human gut bacterium *Bacteroides eggerthii*

Cathleen Kmezik, Daniel Krska, Scott Mazurkewich, Johan Larsbrink

Table S1: Amino acid sequence-based predictions of glycoside hydrolases and carbohydrate esterases present in PUL 27 of *B. eggerthii* based on PDB entries.

Locus tag	Enzyme family	Best hit in PDB	PDB accession	sequence coverage [%]	sequence identity [%]
HMPREF1016_02151	GH35	*Cellvibrio japonicus* β-galactosidase Bgl35A	4D1I	79	33
HMPREF1016_02154	GH43_1	β-xylosidase from uncultured organism	4MLG	99	79
HMPREF1016_02155	GH67	*C. japonicus* α-D-glucuronidase	1GQ1	89	48
HMPREF1016_02157	GH115	*Bacteroides ovatus* α-glucuronidase	4C90	99	72
HMPREF1016_02159	CE15	*Thermothelomyces thermophilus* 4-*O*-methyl glucuronoyl esterase	4GAG	93	39
	GH8	*Paenibacillus barengoltzii* Rex	5YXT	98	43
HMPREF1016_02160	GH97	*Bacteroides thetaiotaomicron* β-arabinopyranosidase	5XFM	95	57
HMPREF1016_02162	GH31	*C. japonicus* α-xylosidase	2XVG	98	36
HMPREF1016_02163	GH43_10	*Bacteroides eggerthii* *Be*GH43/FAE	6MLY	100	100
HMPREF1016_02164	CBM48-CE1	*Bacteroides intestinalis* acetyl xylan esterase	6NE9	99	74
HMPREF1016_02167	GH5_21	no significant similarity found	-	-	-
HMPREF1016_02168	GH10	*B. intestinalis* *Bi*Xyn10A	4MGS	20	33
HMPREF1016_02174	GH43_12	*Geobacillus thermoleovorans* β-1,4-xylosidase	5Z5D	88	38

Table S2: Primers used to amplify genes of *B. eggerthii*. The nucleotide sequences are written from 5' to 3' direction.

Construct		DNA sequence
*Be*CE15A	F	CTTCCAGGGCCATAGTGACAAAGAATGTGCTAATGACAACTTTCGTG
	R	TGGTGGTGCTCGAGTCTAAGATAAACGTTCTATCTCATTTGCCCAAG
*Be*Rex8A	F	CTTCCAGGGCCATAGTGAAGAGAGGCTGCCCTATACCAAAGGT
	R	TGGTGGTGCTCGAGTCTATCCTTGAGGAAATATAATCCGATAGTTCCCG
*Be*CE15A F200R	F	TGTTCACGTGCCGGTAAAATGGCATTGTTTG
	R	ACCGGCACGTGAACAACCGGATACTG
*Be*CE15A F200Y	F	GCAGTATCCGGTTGTTCATATGCCGGTAAAATGGCATTG
	R	CAATGCCATTTTACCGGCATATGAACAACCGGATACTGC
*Be*Rex8A R257A	F	CACATTGCTTGGCAGTAAAGCGGTTATAGGAGATGCTTTTC
	R	GAAAAGCATCTCCTATAACCGCTTTACTGCCAAGCAATGTG

Each protein was cloned using primer pairs consisting of forward primers (F) and reverse primers (R). Gray highlights mark overhangs homologous to the cloning site in pET28a-TEVc and underlining marks the introduction of mutations. The mutation from adenosine to thymine (*Be*CE15A-F and *Be*CE15A-Rex8A-F) was introduced to reduce the number of adenosine residues in the primer, without changing the transcribed amino acid (alanine). In *Be*CE15A-R a mutation was introduced to replace a glycine residue with a stop codon. The full-length *Be*CE15A-Rex8A, lacking the predicted signal peptide (residues 1-31), was cloned using the primers *Be*CE15A-F and *Be*Rex8A-R.

Table S3: Multistep gradient applied for the separation of xylooligosaccharides ranging from xylose to xylohexaose.

Step/ gradient	Time	dH_2O [%]	300 mM NaOH [%]	1 M sodium acetate [%]
step 1	-5 to 0 min	85	15	0
injection at 0 min				
gradient 1	0 to 10 min	85 to 67	15 to 33	0
gradient 2	10 to 15 min	67 to 57	33	0 to 10
gradient 3	15 to 17.5 min	57 to 0	33	10 to 67
step 2	17.5 to 22.5 min	0	33	67
step 3	22.5 to 25 min	85	15	0

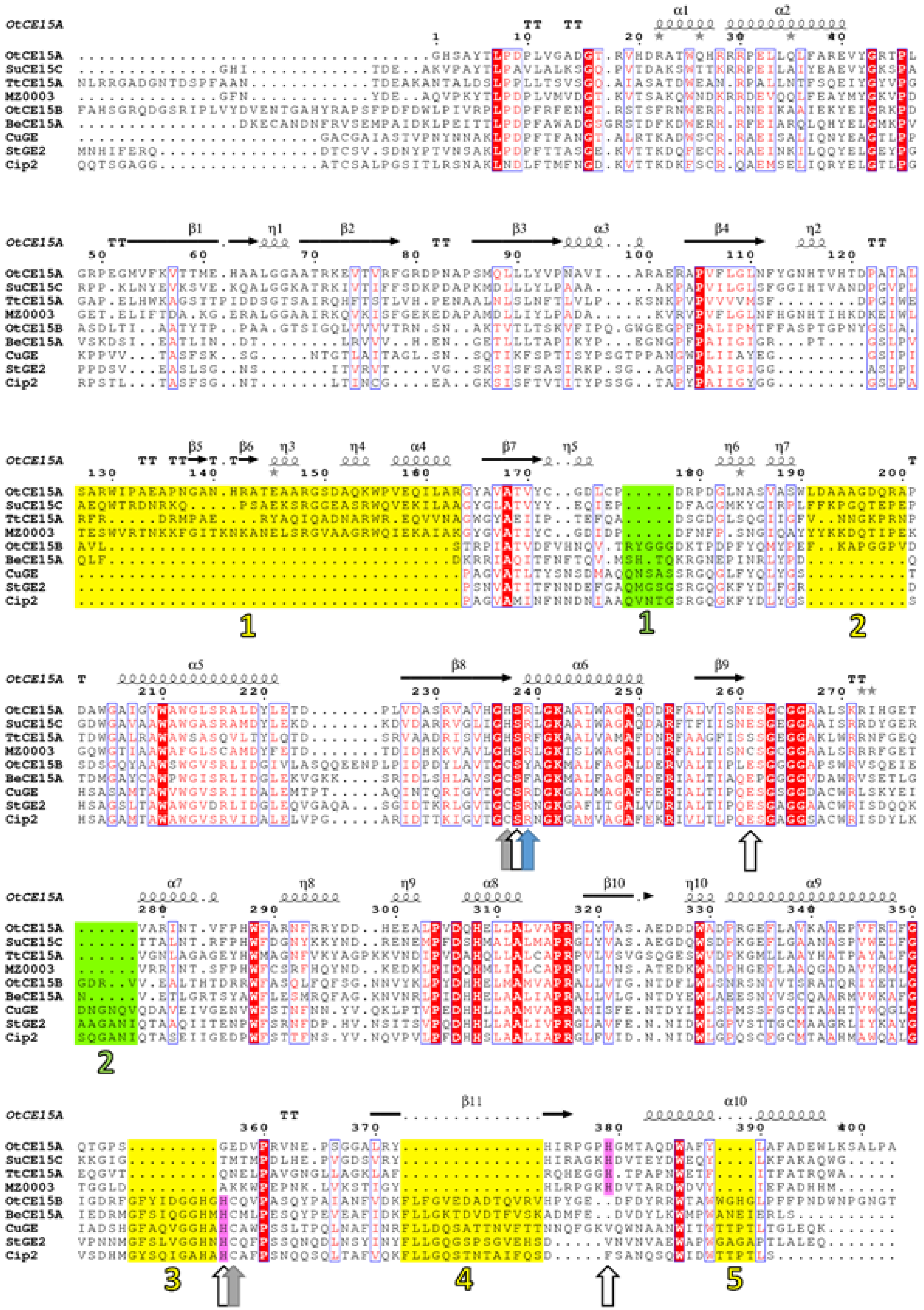

Figure S1: Sequence-based alignment of *Be*CE15A. The secondary structural elements of *Ot*CE15A [1], a glucuronoyl esterase from *Opitutus terrae* (PDB accession 6SYU) served as template. While *Be*CE15A and *Ot*CE15B [2] are of bacterial origin, they exhibit structural features more closely related to fungal GEs and are here referred to as "fungal-like". Catalytic residues are marked with white arrows.

The catalytic histidine is additionally marked by pink highlights. Unfortunately, the alignment parameters did not allow for this residue to be placed in the same column for classical bacterial GEs and fungal/ fungal-like GEs. The blue arrow marks the mostly conserved arginine in close proximity to the catalytic serine, that is substituted by phenylalanine in *Be*CE15A. The gray arrows mark the cysteine residues forming a disulfide bridge common in fungal and fungal-like GEs. Further, the residue marked by a gray arrow in close proximity to the catalytic serine appears to be conserved in the typical bacterial GEs as histidine and in fungal/fungal-like GEs as cysteine. Yellow highlights mark regions of insertion that are not present in classic bacterial GEs. Green highlights mark regions of insertion that are not present in fungal and fungal-like GEs. Further characterized GEs included in this alignment are from *Solibacter usitatus* (*Su*CE15C; [2]), *Teredinibacter turnerae* (*Tt*CE15A; [3]), a bacterial marine metagenome sample (MZ0003; [4]), *Cerrena unicolor* (*Cu*GE; [5]), *Sporotrichum thermophile* (StGE2; [6]) and *Hypocrea jecorina* (Cip2; [7]). The alignment was created in Clustal Omega [8] and visualized using Espript 3.0 [9].

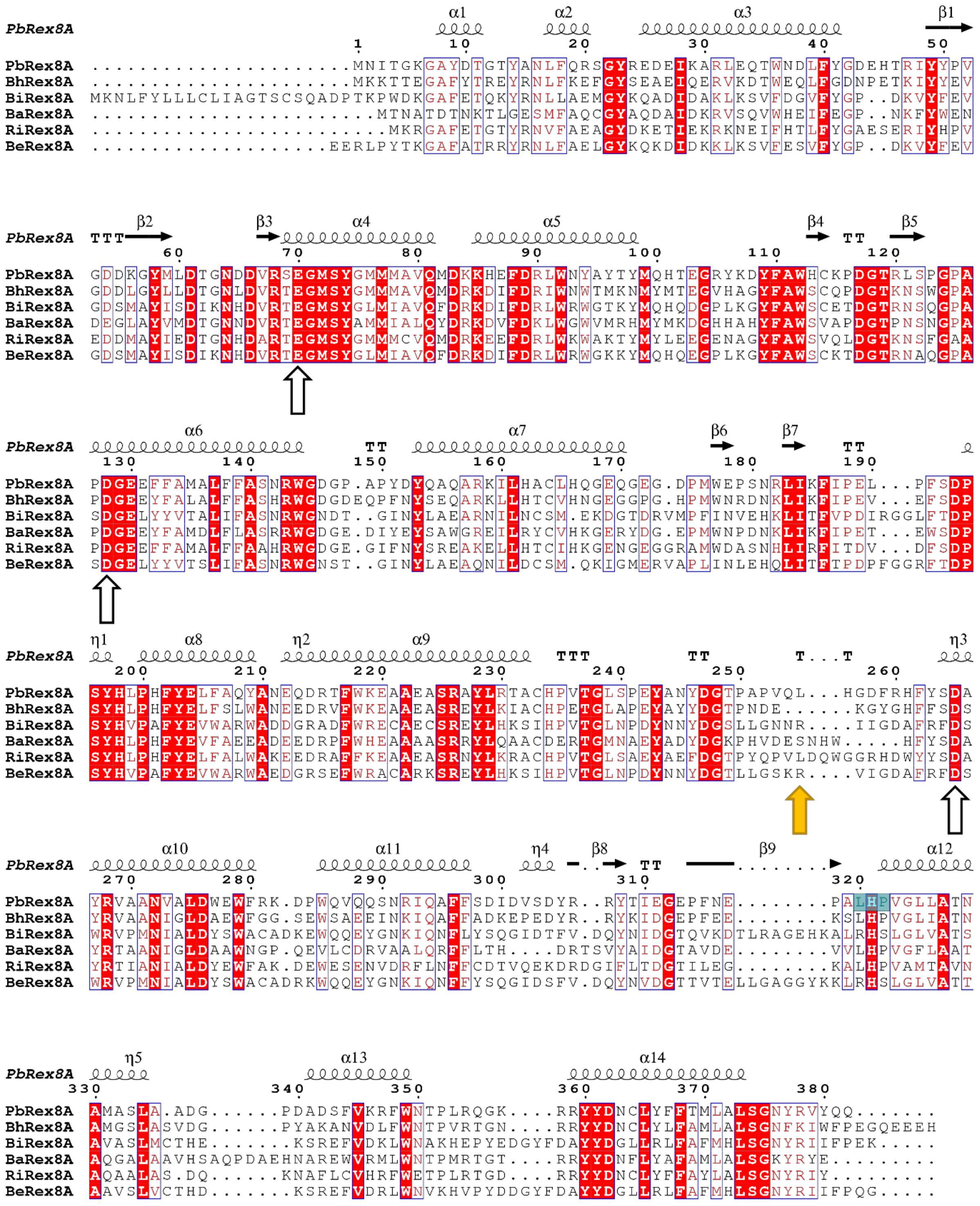

Figure S2: Sequence-based alignment of *Be*Rex8A. The secondary structural elements of *Pb*Rex8A [10], a reducing-end xylose-releasing *exo*-oligoxylanase from *Paenibacillus barcinonensis* BP-23 (PDB accession 6SRD) served as template. Catalytic residues are marked with white arrows, arginine residue 670 is marked by a yellow arrow and the Leu320-His321-Pro322 loop is marked in cyan. Further characterized Rex included in the alignment are from *Bacillus halodurans* C-125 (*Bh*Rex8A; [11]), *Bacteroides intestinalis* DSM 17393 (*Bi*Rex8A; [12]), *Bifidobacterium adolescentis* ATCC 15703 (*Ba*Rex8A; [13]) and *Roseburia intestinalis* L1-82 (*Ri*Rex8A; [14]). The alignment was created in Clustal Omega [8] and visualized using Espript 3.0 [9].

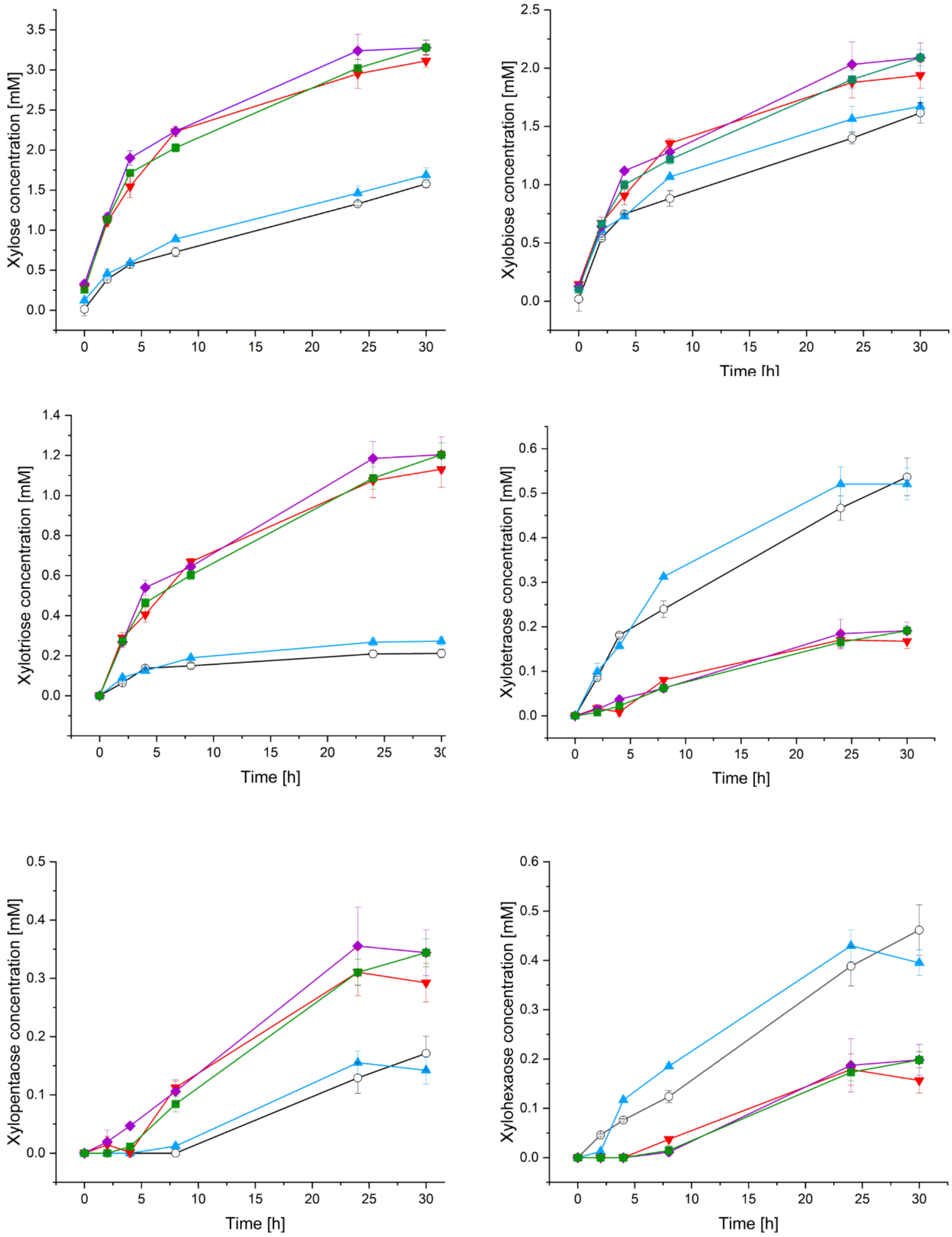

Figure S3: Enzymatic xylooligosaccharide (XO) release from corn cob. Samples were incubated for 30 hours with: Xyn11A (control; black open circles), Xyn11A + *Be*CE15A (blue triangle), Xyn11A + *Be*Rex8A (red triangle), Xyn11A + *Be*CE15A-Rex8A (purple diamond) and Xyn11A + *Be*CE15A + *Be*Rex8A (green square). XO concentrations were quantified using HPAEC-PAD. Data represent averages of triplicate experiments with standard error of mean.

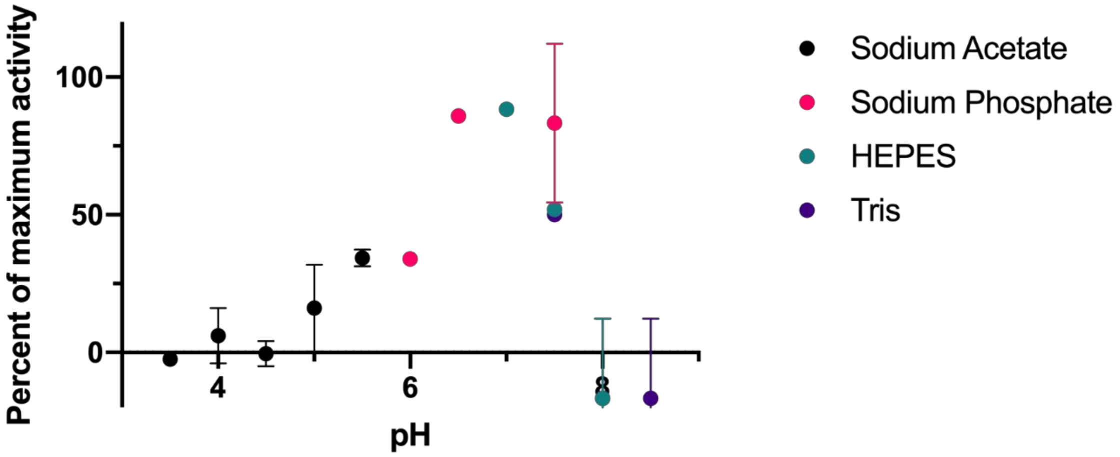

Figure S4: pH dependency of the GE domain of *Be*CE15A-Rex8A. Measurements were taken in sodium acetate buffer (black), sodium phosphate buffer (pink), HEPES buffer (teal) and Tris buffer (purple). Higher and lower pH levels were unfeasible to test with the model substrates used. Seemingly negative activity is a result of background activity.

References

1. Mazurkewich, S., Poulsen, J. N., Lo Leggio, L., and Larsbrink, J. Structural and biochemical studies of the glucuronoyl esterase *Ot*CE15A illuminate its interaction with lignocellulosic components. *Journal of Biological Chemistry* **294**, 19978-19987 (2019).
2. Arnling Bååth, J. *et al.* Biochemical and structural features of diverse bacterial glucuronoyl esterases facilitating recalcitrant biomass conversion. *Biotechnology for Biofuels* **11**, 1-14 (2018).
3. Arnling Baath, J., Mazurkewich, S., Poulsen, J. N., Olsson, L., Lo Leggio, L., and Larsbrink, J. Structure-function analyses reveal that a glucuronoyl esterase from *Teredinibacter turnerae* interacts with carbohydrates and aromatic compounds. *Journal of Biological Chemistry* **294**, 6635-6644 (2019).
4. De Santi, C., Gani, O. A., Helland, R., and Williamson, A. Structural insight into a CE15 esterase from the marine bacterial metagenome. *Scientific Reports* **7**, 1-10 (2017).
5. Ernst, H. A. *et al.* The structural basis of fungal glucuronoyl esterase activity on natural substrates. *Nature Communications* **11**, 1-12 (2020).
6. Charavgi, M.-D., Dimarogona, M., Topakas, E., Christakopoulos, P., and Chrysina, E. D. The structure of a novel glucuronoyl esterase from *Myceliophthora thermophila* gives new insights into its role as a potential biocatalyst. *Acta Crystallographica Section D: Biological Crystallography* **69**, 63-73 (2013).
7. Pokkuluri, P. R. *et al.* Structure of the catalytic domain of glucuronoyl esterase Cip2 from *Hypocrea jecorina*. *Proteins* **79**, 2588-2592 (2011).
8. Madeira, F., Park, Y. M., Lee, J., Buso, N., Gur, T., Madhusoodanan, N., Basutkar, P., Tivey, A. R. N., Potter, S. C., Finn, R. D., and Lopez, R. The EMBL-EBI search and sequence analysis tools APIs in 2019. *Nucleic Acids Research* **47**, W636-W641 (2019).
9. Robert, X., and Gouet, P. Deciphering key features in protein structures with the new ENDscript server. *Nucleic Acids Research* **42**, W320-W324 (2014).
10. Jimenez-Ortega, E., Valenzuela, S., Ramirez-Escudero, M., Pastor, F. J., and Sanz-Aparicio, J. Structural analysis of the reducing-end xylose-releasing exo-oligoxylanase Rex8A from *Paenibacillus barcinonensis* BP-23 deciphers its molecular specificity. *The FEBS Journal* **287**, 5362-5374 (2020).

11. Honda, Y., and Kitaoka, M. (2004) A family 8 glycoside hydrolase from *Bacillus halodurans* C-125 (BH2105) is a reducing end xylose-releasing exo-oligoxylanase. *Journal of Biological Chemistry* **279**, 55097-55103
12. Hong, P. Y., Iakiviak, M., Dodd, D., Zhang, M., Mackie, R. I., and Cann, I. (2014) Two new xylanases with different substrate specificities from the human gut bacterium *Bacteroides intestinalis* DSM 17393. *Applied and Environmental Microbiology* **80**, 2084-2093
13. Lagaert, S., Van Campenhout, S., Pollet, A., Bourgois, T. M., Delcour, J. A., Courtin, C. M., and Volckaert, G. (2007) Recombinant expression and characterization of a reducing-end xylose-releasing exo-oligoxylanase from *Bifidobacterium adolescentis*. *Applied and Environmental Microbiology* **73**, 5374-5377
14. Leth, M. L., Ejby, M., Workman, C., Ewald, D. A., Pedersen, S. S., Sternberg, C., Bahl, M. I., Licht, T. R., Aachmann, F. L., and Westereng, B. (2018) Differential bacterial capture and transport preferences facilitate co-growth on dietary xylan in the human gut. *Nature Microbiology* **3**, 570-580
